# Pilot Study of Growth Factors in Colostrum: How Delivery Mode and Maternal Health Impact IGF-1, EGF, NGF, and TGF-β Levels in Polish Women

**DOI:** 10.3390/nu17081386

**Published:** 2025-04-20

**Authors:** Paweł Paśko, Jadwiga Kryczyk-Kozioł, Paweł Zagrodzki, Ewelina Prochownik, Martyna Ziomek, Ryszard Lauterbach, Hubert Huras, Magdalena Staśkiewicz, Justyna Dobrowolska-Iwanek

**Affiliations:** 1 Department of Food Chemistry and Nutrition, Faculty of Pharmacy, Jagiellonian University Medical College, Medyczna 9, 30-688 Cracow, Poland; p.pasko@uj.edu.pl (P.P.); jadwiga.kryczyk@uj.edu.pl (J.K.-K.); pawel.zagrodzki@uj.edu.pl (P.Z.); ewelina.gajdzik@uj.edu.pl (E.P.); martyna.ziomek@student.uj.edu.pl (M.Z.); 2 Department of Neonatology, Jagiellonian University Hospital, Kopernika 23, 31-501 Cracow, Poland; ryszard.lauterbach@uj.edu.pl; 3 Department of Obstetrics and Perinatology, Jagiellonian University Hospital, Kopernika 23, 31-501 Cracow, Poland; hubert.huras@uj.edu.pl (H.H.); magorecka@su.krakow.pl (M.S.)

**Keywords:** nutrition, growth factors, colostrum, gestational diabetes, hypothyroidism, maternal health

## Abstract

Background: Breast milk is the most important nutrition for newborns. Growth factors such as insulin-like growth factor 1 (IGF-1), epidermal growth factor (EGF), transforming growth factor-β (TGF-β), and nerve growth factor (NGF) are among its components that play pivotal roles in neonatal development, immune system priming, and gastrointestinal maturation. This study examined the effects of gestational diabetes mellitus (GDM), maternal hypothyroidism, and method of delivery on the concentrations of these factors in colostrum collected at three distinct postpartum time points. Methods: A group of 39 women was included, 20 of whom gave birth vaginally, whereas caesarean section was performed in 19 patients. A total of 18 volunteers were diagnosed with GDM, and 17 suffered from hypothyroidism. Colostrum samples were collected from the volunteers in the first 3 days after birth under hospital conditions. Growth factors like IGF-1, EGF, NGF, and TGF-β were measured in the samples using commercial immunoenzymatic assays. Results: No significant differences were observed in the values of these parameters between the groups of women (with GDM or hypothyroidism and healthy, as well as giving birth naturally and by caesarean section). In addition, the growth factors exhibited good stability within the first few postpartum days (CVs for all studied parameters: in the range of 0.7–5.0%). Conclusions: The pregnancy disorders that were properly controlled and treated by specialists appeared not to affect the levels of the analyzed growth factors—just like the type of delivery and the day of colostrum collection.

## 1. Introduction

Gestational diabetes mellitus (GDM) may affect about 14% of pregnant women worldwide [1] and is therefore a significant public health problem. Maternal age, overweight/obesity before pregnancy, first pregnancy, and family history of diabetes are among the factors that increase the risk of it [2]. Importantly, GDM poses a risk of disorders in the course of pregnancy (e.g., preterm birth, pre-eclampsia) in the development of the child (e.g., macrosomia, obesity, or cardiovascular risk) but also in the woman after delivery (e.g., risk of type 2 diabetes and cardiovascular disease) [3]. Besides GDM, hypothyroidism is an example of another disorder quite common among pregnant women [4], which may also be relevant to the child’s development [5].

Growth and immune-regulatory factors present in maternal blood and breast milk, such as IGF-1, EGF, and TGF-β, are pivotal in fetal growth and neonatal development. These factors, abundant in colostrum and breast milk, play crucial roles in priming the neonatal immune system, supporting gastrointestinal maturation, and driving postnatal growth, making them vital not only during fetal life but also throughout neonatal adaptation [6].

In a healthy pregnancy, maternal IGF-1 and TGF-β levels undergo tightly regulated changes that support placental function and optimize the fetal nutrient supply, directly influencing the fetal size and birth weight. However, in pregnancies complicated by metabolic or endocrine disorders such as GDM or maternal hypothyroidism, these regulatory mechanisms are significantly altered. Such changes can disrupt the placental physiology, influence fetal growth, and affect neonatal outcomes. In diabetic pregnancies, IGF-1 levels are typically elevated, with concurrent alterations in insulin-like growth factor binding proteins (IGFBPs), which modulate IGF-1 bioavailability. These changes, along with the upregulation of placental nutrient transport pathways (e.g., mechanistic target of rapamycin—mTOR), often lead to excessive fetal growth or macrosomia [7]. Additionally, elevated levels of TGF-β and vascular endothelial growth factor (VEGF) in diabetic pregnancies have been associated with adaptive but often pathological placental changes, such as increased vascularization, further affecting nutrient transfer and growth regulation [8]. Notably, lower NGF levels have been observed in the infants of diabetic mothers, particularly those with intrauterine growth restriction (IUGR), suggesting that maternal diabetes may impair fetal neurodevelopment through altered NGF dynamics [9].

Conversely, maternal hypothyroidism is associated with reduced IGF-1 levels at the maternal–fetal interface, a factor closely linked to IUGR. Thyroid hormone deficiency disrupts the endocrine environment required for IGF-1 synthesis, compromising fetal tissue growth and maturation. In cases of hypothyroidism co-occurring with IUGR, reductions in IGF-1 levels are especially pronounced, exacerbating growth limitations [10]. Interestingly, while maternal thyroid function in IUGR pregnancies is not distinctly altered aside from elevated iodine blood levels, higher triiodothyronine (T3) concentrations in the cord blood of growth-restricted fetuses suggest a compensatory mechanism. However, no research to date has explored the impact of maternal thyroid dysfunction on NGF levels, leaving a critical gap in understanding its role in the neurodevelopmental pathways.

The benefits of breast milk feeding to the infant have already been well-evidenced; therefore, the World Health Organization recommends exclusive breastfeeding for the first 6 months of life [11]. Colostrum and breast milk are not merely nutritional resources but are also rich in immune-regulatory factors critical for neonatal development. Immediately after birth, neonates receive high concentrations of secretory immunoglobulin A (IgA) and other antibodies, which play a pivotal role in establishing passive immunity, via colostrum. These antibodies offer a first line of defense against pathogens by binding to microbial antigens in the gut and preventing their translocation across the mucosal barrier. In addition to IgA, colostrum and breast milk contain a spectrum of bioactive molecules—including cytokines, chemokines, growth factors, lactoferrin, and lysozyme—that collectively contribute to the maturation of the mucosal immune system and the formation of the intestinal microbiota. This humoral arm of immunity not only affords immediate protection but also influences the subsequent development of the infant’s adaptive immune responses, thereby setting the stage for long-term immune homeostasis. Such interplay between the maternal immune factors and the neonatal immune system is essential for the gradual establishment of an effective yet balanced immune defense during early life [12,13,14].

It has been hypothesized that selected endocrine disorders during pregnancy, as well as the mode of delivery, may influence the concentrations of growth and immune-regulatory factors in colostrum. Therefore, in this study, we assessed the contents of growth factors in colostrum in the first days of lactation, taking into account selected variables such as gestational diabetes, hypothyroidism in pregnancy, and type of delivery. This approach could make a valuable contribution to the understanding of maternal and neonatal health in complex pregnancies.

## 2. Materials and Methods

### 2.1. Study Group Design and Sample Collection

Colostrum samples were collected from women living in the city of Kraków (population: approximately 800,000), located in southern Poland. All patients gave birth in the University Hospital in Kraków. The inclusion and exclusion criteria for the eligible patients were as described in a previous article [15] and are shown in Table 1.

**Figure 1:**
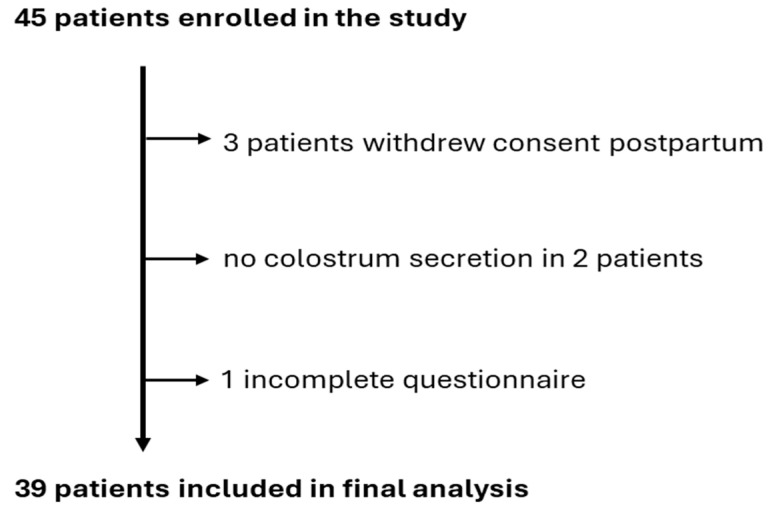
Flowchart of participants throughout this study.

Of the 45 patients initially enrolled in this study, three withdrew their consent postpartum. In two cases, colostrum samples were not collected successfully, and one participant submitted an incomplete questionnaire, which rendered the data unusable. Finally, this study included 39 patients (Figure 1), aged 20–42 years, with pre-delivery body weights of 59–120 kg and heights of 155–182 cm.

A total of 18 participants were diagnosed with GDM. These individuals were managed under the supervision of a specialist diabetologist and received individualized therapeutic protocols, primarily involving the administration of various formulations of human or analogue insulin, alongside adherence to a diabetic diet. Pharmacological intervention was not required for two participants, who were managed with dietary measures alone. In addition, 17 participants were diagnosed with hypothyroidism during pregnancy and received specialist endocrine care, including levothyroxine replacement therapy. With regard to comorbidities, arterial hypertension was identified in five participants, all of whom were treated with methyldopa in accordance with current clinical guidelines, including those of the American College of Obstetricians and Gynecologists (ACOG) and the European Society of Cardiology (ESC), which recommend methyldopa as a first-line agent for the management of hypertension during pregnancy. Prophylactic antithrombotic therapy was administered to seven patients and included agents such as acetylsalicylic acid (an antiplatelet agent) and enoxaparin sodium (a low-molecular-weight heparin with anticoagulant properties), in line with current recommendations for the prevention of thromboembolic complications. Etamsylate, a hemostatic agent, was used in select cases to manage bleeding risk. Renal dysfunction was observed in two participants, and one patient had a documented history of deep-vein thrombosis of the lower extremities. All participants declared regular intake of supplements during pregnancy with the recommendations of the Polish Society of Gynecologists and Obstetricians [16]. The patients who were not diagnosed with gestational diabetes received typical balanced diets. Among the volunteers, 20 gave birth by the vaginal route and 19 by caesarean section. Regardless of the mode of delivery, all patients received oxytocin for the following purposes: (i) to induce or augment labor (vaginal delivery) or (ii) to promote effective uterine contraction postpartum, thereby preventing postpartum hemorrhage (both vaginal and caesarean deliveries). The patients who underwent caesarean section additionally received prophylactic antibiotic therapy, most commonly a cephalosporin or an aminopenicillin, in accordance with the current obstetric and surgical guidelines for infection prevention. In five patients who underwent vaginal delivery, amoxicillin was administered in the postpartum period due to the occurrence of complications.

### 2.2. Sample Preparation and Validation Parameters

Colostrum samples were collected up to the third day after birth with the help of qualified medical personnel. The colostrum samples were dispensed into Eppendorf-type tubes and frozen at −70 °C. On the day of analysis, after thawing on ice, each sample was centrifuged at 21 °C for 10 min at 4000 rpm. The supernatant was then collected for determination of the following parameters: IGF-1, EGF, NGF, and TGF-β. Parameter determinations were made using commercial immunoenzymatic assays (Human INF-1 ELISA KIT—E0103Hu; Human EGF ELISA KIT—E0144Hu; Human NGF ELISA KIT—E2102Hu; Human TGF-β ELISA KIT—E3051Hu) purchased from Bioassay Technology Laboratory (Zhejiang, China). For each patient, three independent colostrum samples were collected and analyzed to ensure measurement reliability. Each assay was performed according to the protocol provided with the test kit. A modular multi-sensing microplate reader, SynergyTM 2 (BioTek Instruments, Winooski, VT, USA), was used to read the absorbance values within 10 min of the administration of the inhibitory solution at a wavelength of 450 nm. The analytical parameters of the ELISA utilized for quantifying the growth factors are displayed in Table 2.

This study was conducted in accordance with the Declaration of Helsinki and approved by the Ethical Committee of Jagiellonian University (No. 1072.6120.142.2023).

### 2.3. Statistical Approach

The distributions of the studied parameters were checked using the Kolmogorov–Smirnov and Shapiro–Wilk tests. To estimate the significance of the differences between the mean values in various subgroups, we used the parametric Student *t*-test for 2 independent samples or analysis of variance in a repeated-measures scheme in cases of examining the variability of parameters on successive days after delivery. The homogeneity of the variance of the tested parameters was checked by Levene’s test. The statistical significance was set for *p* < 0.05. These analyses were performed using STATISTICA PL v.13 (TIBCO Software Inc., Palo Alto, CA, USA).

## 3. Results

**Figure 2:**
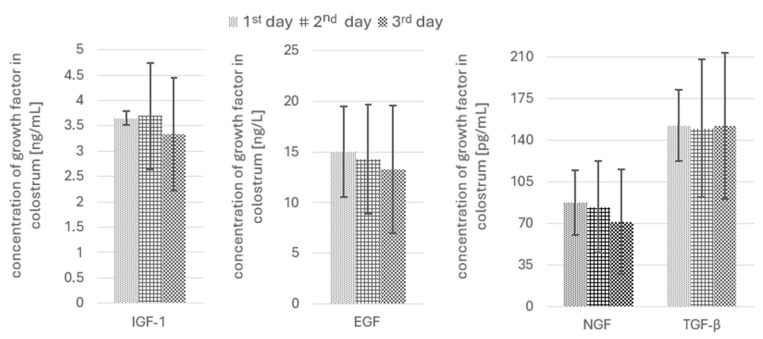
The concentrations of IGF-1, EGF, NGF, and TGF-β in colostrum samples collected on the first, second, and third day of lactation. Results expressed as means ± SD. No statistical differences among obtained results; *p* > 0.05.

The results of the growth and immune-regulatory factor determinations in the colostrum were divided into 6 subgroups of patients, i.e., without gestational diabetes mellitus/with gestational diabetes mellitus (summarized in Table 3); without hypothyroidism in pregnancy/with hypothyroidism in pregnancy (summarized in Table 4); and giving birth by the vaginal route/giving birth by caesarean section (summarized in Table 5). The outcomes were expressed as means and standard deviation, medians and range of values (minimum–maximum), and lower and upper quartiles. The distributions of all examined parameters met the normality criteria. The Kolmogorov–Smirnov test d score ranged from 0.087 (*p* = 0.912) to 0.151 (*p* = 0.321), and the Shapiro–Wilk test score ranged from 0.944 (*p* = 0.057) to 0.981 (*p* = 0.746). There were no significant differences (*p* > 0.05) in the concentrations of all parameters (i.e., IGF-1, EGF, NGF, and TGF-β) between the participants meeting the following criteria: presence/absence of gestational diabetes mellitus; presence/absence of hypothyroidism in pregnancy; and type of delivery (natural/caesarean section) in well-controlled maternal conditions. The postpartum day at which each sample was taken (i.e., the first, second, and third) also had no significant effect (*p* > 0.05) on the levels of the tested parameters (Figure 2). Nevertheless, a decreasing trend among the mean values of these parameters was observed between the 1st and 3rd day, i.e.,: 8.8%—IGF-1; 11.4%—EGF; 18.5%—NGF, and 0.1%—TGF-β.

## 4. Discussion

### 4.1. Diabetes and Growth Factors in Colostrum

To date, only a few studies have investigated the relationship between diabetes during pregnancy and growth factors in colostrum—mainly focused on IGF-1. Mohsen et al. [17] evaluated the concentrations of this factor in the breast milk of diabetic mothers and in the sera of their newborns, obtaining statistically higher values compared to the control group. Moreover, they revealed a positive correlation between milk IGF-1 levels and various anthropometric measurements, except for the baby’s length at birth. Galante et al. [18] reported a dependence between IGF-1 concentrations and weight gain during pregnancy. Additionally, it was found that the infant’s gender was involved in the association between gestational diabetes and the level of this parameter in milk. It is possible that changes in the composition of breast milk are affected not only by maternal and infant characteristics but also by pathophysiological factors. Nevertheless, in our study, there were no significant variations in the levels of the assayed growth factors, including IGF-1 (Table 3). Similarly, in another study conducted in Poland, there were no significant differences in the concentration of this parameter in relation to maternal hyperglycemia. This lack of variation might be due to the effective management of GDM during pregnancy, as all participants were under specialized medical care [19].

### 4.2. Hypothyroidism and Growth Factors in Colostrum

We did not observe significant differences in the analyzed parameters in the colostrum of the mothers with hypothyroidism during pregnancy compared to the healthy ones (Table 4). Attempts to interpret these results would be highly problematic due to the lack of available studies directly linking this thyroid disorder with changes in the growth factors in colostrum. However, Chen et al. [20] noted that gestational hypothyroidism (G-HypoT) may alter the composition of colostrum whey protein. Specifically, it decreases metabolic and cell-structural proteins while increasing immune-related proteins, which could reflect the health statuses of both mothers and infants. Interestingly, Jin et al. [21] also reported significantly lower protein content in the mature milk of G-HypoT women compared to the control group.

The absence of significant differences in colostrum composition among women with well-controlled GDM or hypothyroidism, as observed in our study, may be attributed to effective medical management mitigating the potential biological impacts of these conditions. However, it is important to recognize that even with treatment, subtle alterations in colostrum composition can occur. For instance, Gámez-Valdez et al. [22] have demonstrated that GDM can influence the immunological profile of colostrum. A study by Avellar et al. [23] found that mothers with GDM exhibited higher levels of pro-inflammatory cytokines, such as IFN-γ, IL-6, and IL-15, in their colostrum, with reduced concentrations of GM-CSF, compared to healthy controls. These findings suggest that GDM may lead to immune alterations in colostrum, potentially affecting neonatal immune development. Regarding hypothyroidism, while direct studies on its impact on colostrum composition are limited, existing research indicates that thyroid dysfunction can alter the nutritional and immunological properties of breast milk. For example, in the above-mentioned study, Jin et al. [21] highlighted that hypothyroidism during pregnancy affects the nutritional composition of human milk, which may influence infant development.

These studies underscore that even with medical management, GDM and hypothyroidism can subtly affect colostrum’s immunological and microbiological composition. Therefore, it is plausible that the lack of significant differences in our study could have resulted from effective treatment regimens mitigating more pronounced alterations. Nonetheless, the potential for subtle changes underscores the need for further research with larger sample sizes and more sensitive analytical methods to fully elucidate these effects.

### 4.3. Delivery Method and Growth Factors in Colostrum

The influence of delivery methods on IGF-1, EGF, NGF, and TGF-β is of considerable interest due to their roles in neonatal development and maternal adaptation. Kociszewska-Najman et al. [24] found that TGF-β2 concentrations in colostrum were higher in mothers who delivered via cesarean section compared with those who delivered vaginally. The TGF-β2 levels were slightly higher in preterm compared with term deliveries (4648 ng/mL vs. 3899 ng/mL). In the cesarean deliveries, the TGF-β2 levels were significantly higher (7429 ng/mL) than in the vaginal deliveries (5240 ng/mL). These findings suggest that the mode of delivery might influence the immune and growth factor compositions of colostrum, particularly for cesarean-delivered infants who may have different microbial exposures. Our results are in opposition to this assumption, as we obtained comparable values for TGF-β in both groups of women (Table 5). Therefore, further studies with larger sample sizes and microbiome profiling are needed. However, it should be emphasized that the aforementioned study measured a specific TGF-β isoform, whereas we assessed the total levels without distinguishing between its subtypes. These discrepancies in the results may also prove the importance of analyzing specific isoforms separately, as they may respond differently to maternal or perinatal factors. In turn, Pawlus et al. [25] observed that the IGF-1 concentrations in the colostrum of women delivering by cesarean section were lower than in those delivering vaginally; however, a significant difference was noted only in comparison with women giving birth at term. In the present study, we also found only slightly lower concentrations of this factor in the colostrum of women delivering by caesarean section compared with mothers giving birth vaginally (Table 5). Thus, it is possible that cesarean section may disrupt the synthesis and release of IGF-1 into the milk in early stages of lactation. Vaginal delivery promotes higher levels of growth factors in colostrum, likely due to the natural physiological and hormonal processes involved in labor. Although cesarean delivery is necessary in certain cases, it may reduce the initial levels of these growth factors. This suggests that nutritional interventions could benefit infants born via cesarean section. Additional research is needed to fully understand these dynamics.

The literature data on EGF and NGF refer mainly to their contents in breast milk depending on the duration of the pregnancy rather than the type of delivery, which proves the novelty of our study but thus makes it difficult to interpret the results (Table 5). Castellote et al. [26] compared the levels of EGF and TGF-β (i.e., TGF-β1 and TGF-β2) in the milk of women who gave birth at term, preterm, and very preterm. Milk samples were collected at three points of lactation as colostrum, transitional milk, and mature milk. The concentration of the EGF in the colostrum was significantly higher compared to the milk from the later stages of lactation regardless of the time of delivery. In addition, negative correlations between EGF and birth weight, as well as gestational age, were noted in the transitional milk, which may indicate a special role for this growth factor in the development of preterm and low-birth-weight infants. Similarly, the TGF-β1 and TGF-β2 concentrations were higher in the colostrum of the mothers giving birth preterm than in the term group. These results suggest that lactogenic compensatory mechanisms might accelerate the development of immature preterm infants only after 30 weeks of gestation.

Dangat et al. [27] reported no significant differences between the NGF levels in colostrum samples between women who were healthy and those with pre-eclampsia (i.e., 283.0 ± 208.3 pg/mL and 273.2 ± 214.7 pg/mL) despite a notably shorter duration of pregnancy (39.2 ± 1.0 and 37.4 ± 2.6 weeks). It is worth pointing out the wide individual variability for this growth factor, which was observed in our study as well (Table 5). Similarly, in a study by Moran et al. [28], duration of pregnancy had no compensatory effect on the concentration of EGF in breast milk, as there were no significant differences in its levels between the groups of women who gave birth extremely preterm (range of gestational age: 27–32 weeks) and at term (range of gestational age: 38–40 weeks). However, it is worth noting that all participants delivered vaginally, which makes it difficult to directly match with our study, where the concentrations of the above-described growth factors were compared between women delivering vaginally and by caesarean section (Table 5).

### 4.4. Time of Colostrum Sampling

TGF-β1 and TGF-β2 are abundant in early breast milk and play a critical role in supporting IgA production in newborns, which is essential for mucosal immunity. Interestingly, elevated levels of these growth factors are associated with a reduced risk of atopic dermatitis in infants [6] and may protect against allergic diseases by fostering immune tolerance [29]. Interestingly, in cases of post-weaning-onset atopic disease, the levels of TGF-β1 and TGF-β2 in the maternal colostrum were higher compared with in pre-weaning-onset cases. Additionally, the concentrations of TGF-β2 and, to a lesser extent, TGF-β1 were higher in the colostrum of mothers whose infants had specific IgA-secreting cells at 3 months in response to at least one dietary antigen tested compared with those whose infants did not exhibit such a response [29]. In other studies conducted in the United Kingdom, Russia and Italy found an inverse correlation between the timing of milk sampling and the levels of growth factors such as hepatocyte growth factor (HGF) and TGF-β1 and TGF-β3 but not TGF-β2. The growth factor levels were significantly higher in colostrum than in mature milk, with the kinetics differing for each parameter. Those studies also indicated that growth factor levels can vary between populations [30]. Interestingly, comparisons between colostrum samples from Burundian and Italian mothers revealed that TGF-β levels tend to be higher in regions with greater microbial exposure, emphasizing the role of environmental factors in modulating the immune components in breast milk [31]. In contrast, Hirata et al. [32] observed no effect of such factors as labor-inducing drugs, the sex of the newborn, breast/nipple problems, or nipple care on the concentrations of TGF-β1 and TGF-β2 in colostrum, or, similarly, the timing of colostrum secretion (pre- or postpartum). The present study likewise showed no differences in the TGF-β concentrations in the colostrum during the first 3 days after delivery (Figure 2). However, it is known that both the EGF and IGF-1 levels in human colostrum decline rapidly in the first few days postpartum. The EGF levels dropped from 105 µg/L to 40 µg/L, while the IGF-1 levels decreased from 25.9 µg/L to 5.6 µg/L within the first four days. This decline stabilized slightly between days 4 and 7 [33]. In our study, the concentrations of these parameters also showed a decreasing trend between the 1st and 3rd day (i.e.,: EGF—11.4% and IGF-1—8.8%) but without statistical significance (Figure 2). 

Lu et al. [34] investigated breast milk samples from lactating mothers from three different regions in China on days 1 (colostrum), 14 (transitional milk), and 42 (mature milk). The concentration of EGF in the breast milk decreased significantly over the lactation period. Notably, the EGF levels decreased with higher intakes of proteins, vegetables, fruits, soy products, and dairy foods, as well as total energy. The concentration of this growth factor was also influenced by the region where the mothers lived. Similarly, in a study conducted in a Japanese population, statistically lower concentrations of EGF were reported at 30 days of lactation (mature milk) compared to 1 day (colostrum) [35]. In turn, Moran et al. [28] noted no effect of lactation duration on the EGF concentrations in breast milk, while the mean level of this growth factor in colostrum was not statistically different compared with that in mature milk. This may indicate the influences of various factors affecting the dynamics of changes in this growth factor during lactation.

In a study by Sinkiewicz-Darol et al. [36], the levels of NGF in milk samples collected between 1 and 3 months of lactation were not significantly different compared with samples between 6 and 12 months; however, positive correlations between this parameter and carbohydrate content as well as energy values were only found for earlier-stage lactation milk. In turn, in mature milk, the concentration of this parameter increased about 1.5-fold (at 1.5 months of lactation) and 2.5-fold (at 3.5 and 6 months of lactation) relative to colostrum. The trend of increasing NGF content with the progress of lactation may indicate its involvement in different stages of infant development and growth [37]. The assessments of the changes in the levels of this growth factor in the above-mentioned studies took into account significantly longer lactation periods, making them impossible to directly compare to our results, in which no significant differences were found in the colostrum collected during the first three days after childbirth (Figure 2). 

Given the relatively small sample size (*n* = 39), our study may have been underpowered to detect subtle differences in growth factor levels. Post-hoc power analysis (α = 0.05, effect size = 0.25) showed a statistical power of 70%, indicating an increased risk of type II error. This means that, for example, the observed 11.4% decrease in EGF concentrations between days 1 and 3 (Figure 2) may reflect a biologically meaningful trend that, however, did not reach statistical significance due to limited sample size.

## 5. Limitations of This Study

The main limitations of this study include the relatively small sample size of the patients, which limited the statistical power, and the fact that this study was conducted at a single hospital, which makes it difficult to generalize the results. Furthermore, we measured the growth factor levels during only the first three days after delivery, thus not being able to assess the potential delayed effects of maternal conditions. Also, this study only roughly takes into account the mother’s diet, breastfeeding habits, and, to an even lesser extent, lifestyle, all of which can affect the composition of colostrum. Thus, future studies that take these methodological limitations into account will provide a more detailed and clinically useful understanding of the influence of maternal conditions and modes of delivery on colostrum composition: in particular, on the concentration of growth factors.

## 6. Conclusions

In summary, this study reaffirms the pivotal role of human colostrum as a source of essential growth factors that drive neonatal development and immune modulation. We have revealed that controlled and treated diabetes mellitus or hypothyroidism during pregnancy, as well as type of delivery, had no effect on the content of the tested growth factors in colostrum (IGF-1, EGF, NGF, TGF-β). Moreover, the contents of all evaluated parameters in breast milk were stable within the first 3 days of lactation. A key limitation of this study was the small sample size. Future research should prioritize larger cohorts to validate these findings across diverse populations, incorporate microbiome analyses, and investigate additional maternal health conditions and dietary influences on colostrum composition. Such insights may help develop targeted interventions to optimize neonatal outcomes, especially in high-risk pregnancies.

## Figures and Tables

**Table 1:** Study participant selection criteria.

Inclusion Criteria	Exclusion Criteria
Female, aged >18 years Physiological pregnancy Term delivery (≥37 weeks of gestation) Singleton pregnancy No fetal genetic disorders Declaration of breastfeeding for at least 3 months Provided informed consent	Use of elimination diet for medical reasons (e.g., phenylketonuria) Use of elimination diet for personal reasons (e.g., veganism, vegetarianism) Cancer diagnosis Urinary or genital tract infection within the past 2 months Multiple pregnancy Use of assisted reproductive technology Active smoker Non-compliance with study protocol Lack of informed consent

**Table 2:** Analytical parameters of the ELISA test.

Parameter	Sensitivity	Calibration Curve Range	Intra-Assay CV * [%]	Inter-Assay CV * [%]
IGF-1	0.058 ng/mL	1.5–24 ng/mL	2.23	3.01
EGF	0.28 ng/L	3.25–120 ng/L	3.58	4.51
NGF	3.48 pg/mL	8–800 pg/mL	2.99	4.31
TGF-β	2.51 pg/mL	32.5–600 pg/mL	4.56	5.17

* CV—coefficient of variation.

**Table 3:** The descriptive statistics for IGF-1, EGF, NGF, and TGF-β concentrations * in colostrum of women without gestational diabetes mellitus ^a^ and with gestational diabetes mellitus ^b^.

Parameter ^^^	Mean ± SD ^#^	Median (Min.–Max.)	LQ–UQ
IGF-1 ^a^ (*n* = 19)	3.46 ± 0.93	3.55 (2.00–5.10)	2.65–4.35
IGF-1 ^b^ (*n* = 17)	3.52 ± 1.13	3.15 (1.85–6.10)	2.65–4.15
EGF ^a^ (*n* = 21)	13.68 ± 5.34	12.55 (5.20–23.30)	10.25–17.65
EGF ^b^ (*n* = 18)	14.30 ± 5.74	13.95 (5.65–24.60)	10.20–16.10
NGF ^a^ (*n* = 20)	75.2 ± 41.0	67.0 (10.2–153.3)	50.0–111.0
NGF ^b^ (*n* = 18)	78.9 ± 37.9	68.1 (28.2–148.2)	52.6–95.8
TGF-β ^a^ (*n* = 20)	149.9 ± 52.4	141.2 (54.8–233.0)	115.8–182.1
TGF-β ^b^ (*n* = 18)	158.0 ± 62.1	150.8 (62.6–283.4)	117.3–193.3

* As (ng/mL) for IGF-1, (ng/L) for EGF, and (pg/mL) for NGF and TGF-β; ^^^ the discrepancy in the number of participants for individual parameters is due to the insufficient amount of biological material; ^#^ no statistical differences between obtained results, *p* > 0.05.

**Table 4:** The descriptive statistics for IGF-1, EGF, NGF, and TGF-β concentrations * in colostrum of women without hypothyroidism in pregnancy ^a^ and with hypothyroidism in pregnancy ^b^.

Parameter ^^^	Mean ± SD ^#^	Median (Min.–Max.)	LQ–UQ
IGF-1 ^a^ (*n* = 21)	3.41 ± 1.01	3.50 (1.85–5.35)	2.50–4.15
IGF-1 ^b^ (*n* = 15)	3.61 ± 1.05	3.40 (2.00–6.10)	3.00–4.35
EGF ^a^ (*n* = 22)	13.86 ± 6.01	13.03 (5.40–24.60)	9.45–17.25
EGF ^b^ (*n* = 17)	14.11 ± 4.84	12.55 (5.20–24.45)	11.85–15.95
NGF ^a^ (*n* = 21)	76.3 ± 42.7	61.1 (10.2–148.2)	45.6–101.0
NGF ^b^ (*n* = 17)	77.8 ± 35.3	69.3 (24.0–153.3)	54.5–93.5
TGF-β ^a^ (*n* = 21)	148.8 ± 61.2	133.5 (54.8–261.2)	94.7–193.3
TGF-β ^b^ (*n* = 17)	159.9 ± 51.4	148.7 (67.7–283.4)	131.3–180.1

* As (ng/mL) for IGF-1, (ng/L) for EGF, and (pg/mL) for NGF and TGF-β; ^^^ the discrepancy in the number of participants for individual parameters is due to the insufficient amount of biological material; ^#^ no statistical differences between obtained results, *p* > 0.05.

**Table 5:** The descriptive statistics for IGF-1, EGF, NGF, and TGF-β concentrations * in colostrum of women giving birth by the vaginal route ^a^ and giving birth by caesarean section ^b^.

Parameter ^^^	Mean ± SD ^#^	Median (Min.–Max.)	LQ–UQ
IGF-1 ^a^ (*n* = 19)	3.67 ± 0.99	3.40 (2.15–6.10)	3.10–4.55
IGF-1 ^b^ (*n* = 17)	3.29 ± 1.03	3.50 (1.85–5.35)	2.45–4.15
EGF ^a^ (*n* = 20)	15.07 ± 5.44	12.55 (5.40–24.60)	11.70–19.18
EGF ^b^ (*n* = 19)	12.81 ± 5.38	12.85 (5.20–24.15)	7.60–17.00
NGF ^a^ (*n* = 20)	84.0 ± 38.8	69.6 (21.9–153.3)	56.3–110.9
NGF ^b^ (*n* = 18)	69.1 ± 38.9	58.7 (10.2–146.1)	36.3–101.0
TGF-β ^a^ (*n* = 20)	159.7 ± 57.4	150.8 (54.8–283.4)	128.0–190.9
TGF-β ^b^ (*n* = 18)	147.1 ± 56.5	143.6 (67.7–261.2)	94.7–192.1

* As (ng/mL) for IGF-1, (ng/L) for EGF, and (pg/mL) for NGF and TGF-β; ^^^ the discrepancy in the number of participants for individual parameters is due to the insufficient amount of biological material; ^#^ no statistical differences between obtained results, *p* > 0.05.

## Data Availability

The original contributions presented in this study are included in the article. Further inquiries can be directed to the corresponding author.
